# Insight into purification of monoclonal antibodies in industrial columns *via* studies of Protein A binding capacity by *in situ* ATR-FTIR spectroscopy†

**DOI:** 10.1039/d1an00985k

**Published:** 2021-07-16

**Authors:** James W. Beattie, Ruth C. Rowland-Jones, Monika Farys, Richard Tran, Sergei G. Kazarian, Bernadette Byrne

**Affiliations:** Department of Life Sciences, Imperial College London South Kensington Campus SW7 2AZ London UK b.byrne@imperial.ac.uk; Department of Chemical Engineering, Imperial College London South Kensington Campus SW7 2AZ London UK s.kazarian@imperial.ac.uk; Biopharm Process Development, GlaxoSmithKline Gunnels Wood Road Stevenage Hertfordshire SG1 2NY UK richard.x.tran@gsk.com ruth.x.rowland-jones@gsk.com monika.x.farys@gsk.com

## Abstract

Therapeutic monoclonal antibodies (mAbs) are effective treatments for a range of cancers and other serious diseases, however mAb treatments cost on average ∼$100 000 per year per patient, limiting their use. Currently, industry favours Protein A affinity chromatography (PrAc) as the key step in downstream processing of mAbs. This step, although highly efficient, represents a significant mAb production cost. Fouling of the Protein A column and Protein A ligand leaching contribute to the cost of mAb production by shortening the life span of the resin. In this study, we assessed the performance of used PrAc resin recovered from the middle inlet, center and outlet as well as the side inlet of a pilot-scale industrial column. We used a combination of static binding capacity (SBC) analysis and Attenuated Total Reflection-Fourier Transform Infrared (ATR-FTIR) spectroscopy to explore the used resin samples. SBC analysis demonstrated that resin from the inlet of the column had lower binding capacity than resin from the column outlet. ATR-FTIR spectroscopy with PLS (partial least square) analysis confirmed the results obtained from SBC analysis. Importantly, *in situ* ATR-FTIR spectroscopy also allowed both measurement of the concentration and assessment of the conformational state of the bound Protein A. Our results reveal that PrAc resin degradation after use is dependent on column location and that neither Protein A ligand leaching nor denaturation are responsible for binding capacity loss.

## Abstract 2

A combination of static binding capacity analysis and ATR-FTIR spectroscopy reveals that loss of binding capacity is not uniform through a used Protein A column and is not due to loss of Protein A ligand.

## Graphical abstract

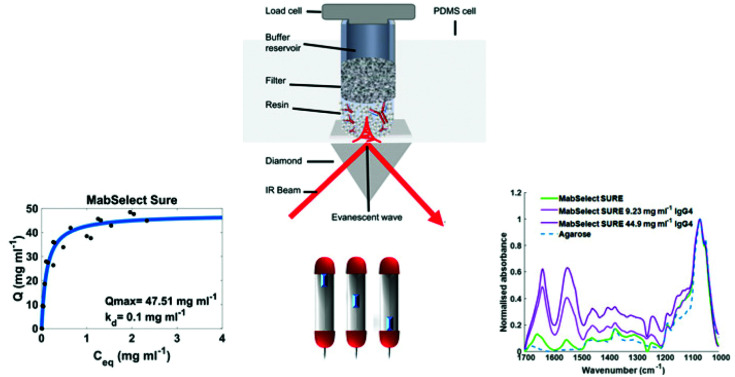

## 1. Introduction

Therapeutic mAbs (Monoclonal Antibodies) are major biopharmaceuticals, making up 22% of the Food and Drug Administration's (FDA) newly approved drugs between 2016–2018.^1^ A total of 550 mAbs were in phase 1 or 2 clinical trials in 2019,^2^ increasing to 743 mAbs in 2020.^3^ In addition, a record 44 cancer and 44 non-cancer targeted mAbs were in FDA and European Medicines Agency (EMA) phase clinical studies as of November 2020.^3^ Currently, therapeutic mAbs available on the market are primarily of the subclass of Immunoglobulin type gamma (IgG).^4^ During 2020 18 mAbs targeting Covid-19 were in phase 2 clinical trials or had been awarded emergency approved use, with the majority being IgGs.^3^ IgG is also the most common antibody isoform present in the body.^5^ The majority of therapeutic mAbs are recombinantly produced in mammalian expression systems, with 60% of mAbs expressed in Chinese hamster ovary cells (CHO).^6^ Due to the recombinant source of mAbs, the presence of small amounts of host cell proteins (HCP) and host cell DNA (HCDNA) in the isolated material is possible. Such contaminants have the potential to trigger a harmful immune response,^7,8^ an undesirable response referred to as immunogenicity.^9^

Various steps are employed in downstream processing of recombinantly produced mAbs in order to reduce HCP and HCDNA to safe levels as well as remove high molecular weight species (HMWS) and culture media components.^5,10^ Safe levels of HCP and HCDNA are recommended at below detectable limits by the FDA^11^ but are typically in the 1 ng mg^−1^ range.^5^ The bulk of contaminating material is removed by Protein A Affinity Chromatography (PrAc).^8^ Protein A reversibly binds to the CH2 and CH3 region (Fc) of mAbs through a combination of hydrogen bonding, salt bridges and hydrophobic interactions.^12^ PrAc is employed in a bind/elute mode with binding of IgG to Protein A performed at neutral pH. The IgG is eluted by decreasing the pH of the column, protonating both Histidine 435 of IgG and Histidine 137 of Protein A causing electrostatic repulsion and elution of the IgG from the column.^13^

PrAc resins have been shown to possess unrivalled purification capabilities, removing 98% of contaminants^8^ and giving a stepwise recovery yield of up to 99.4%.^14^ Protein A chromatography does, however, account for the majority of downstream processing costs, due to the high cost and lifetime degradation of the resin.^15,16^ Downstream processing is responsible for 80% of overall mAb production costs.^15^

Lifetime degradation is attributed in part to irreversible binding of contaminants which may reduce Protein A ligand accessibility. Although the precise nature of these contaminants is unclear, it has been shown that null-cell culture fluid causes less fouling than mAb containing culture fluid^17^ and that HCPs accumulate on the Protein A resin after repeated cycles of purification.^18^

An additional reported cause of lifetime degradation is the harsh alkaline cleaning in place (CIP) procedures used to remove tightly associated contaminant molecules. For every three rounds of Protein A purification performed, one CIP cycle is carried out. CIP protocols usually rely on high concentrations of NaOH (up to 0.5 M),^19^ with trace amounts of Protein A detected in the CIP eluant.^20,21^ To minimize protein A leaching and extend column lifespan, agarose-based resins using engineered Protein A ligands have been developed. MabSelect SuRe, for example, utilizes a Protein A ligand with a modified binding domain B, engineered to be more alkaline resistant.^21^ MabSelect SuRe retains half its binding capacity even after 10 hours of exposure to 0.16 M NaOH.^20^ Boulet *et al.* have shown that the MabSelect SuRe Protein A ligand undergoes denaturation at 1.60 M NaOH but that proteolysis only occurs in extremely harsh conditions, such as 6.46 M NaOH,^20^ a much higher concentration than used for column cleaning.

Whilst it is well known that PrAc resins suffer loss of binding capacity over time, one thing that is not well characterized is whether the loss in binding capacity is homogeneous throughout a Protein A column. A better understanding of this has the potential to make resin use more efficient and thus cut costs associated with mAb purification. Here we used a combination of static binding capacity (SBC) analysis and Attenuated Total Reflection-Fourier Transform Infrared (ATR-FTIR) spectroscopy to explore the performance, ligand density and secondary structure of both Protein A ligand and mAb on MabSelect SuRe samples obtained from a used Protein A column. FTIR spectroscopy is a versatile analytical tool that can analyse the chemical composition of samples in virtually any state. FTIR spectroscopy is a non-destructive, label-free method which can detect multiple components in a system simultaneously. For example, in this study agarose, Protein A ligand, solvent and IgG are all detected in an analysed sample of PrAc resin. The molecular vibrations within a sample absorb mid-infrared radiation of specific frequencies resulting in an energy change, this absorption results in spectral bands at specific wavenumbers making up an individual chemical footprint. ATR allows for the probing of a sample layer of up to 6 μm thickness adjacent to the surface of the IRE (internal reflection element) crystal^22^ overcoming the issue of strong water absorption in the mid-IR range beyond this depth. Previous work from our groups has applied this technique to assessment of the effects of prolonged CIP exposure by immunoaffinity resins,^20^ to monitoring the purification of mAbs in-column^23^ and mAb aggregation.^24,25^ In a measured mid-IR absorption spectrum, prominent spectral features such as the amide I and amide II bands found at 1600 cm^−1^–1700 cm^−1^ and 1520 cm^−1^–1600 cm^−1^ respectively are present for proteins. The exact position of peaks and shoulders within the amide bands are dependent on the protein secondary structure present,^26^ thus the amide bands are extremely important when characterizing proteins.^24,25,27–29^ Partial least squares (PLS) analysis of our spectroscopic data showcased ATR-FTIR spectroscopy as a simple and effective method of predicting performance of affinity resin for mAb capture and exhibiting additional molecular information of measured samples when compared to traditional OD_280 nm_ based static binding capacity assays. Our findings show that the highest losses in binding capacity are experienced at the Protein A resin column inlet and there is a gradual reduction in binding capacity loss through the length of the column. The loss of binding capacity is not due to a reduction in the amount or conformation of the Protein A ligand bound to the resin in the column but is likely due to irreversible binding of mAbs or HCP fouling within the porous matrix of the Protein A resin.

## 2. Experimental

### 2.1 IgG4 preparation

A Glutamine Synthetase Chinese Hamster Ovary (GS-CHO) cell line (Lonza Biologics, Basel, Switzerland) was used to express Chimeric mAb B72.3 (IgG4). Cultures were maintained in CD-CHO medium (ThermoFisher, UK) supplemented with l-methionine sulphoximine (Merck, Gillingham, UK) at 8% CO_2_ humidified air at a temperature of 36.5 °C. The Chimeric cB72.3 IgG4 was secreted into the cell culture supernatant (CCS). Following protein expression, the CCS was harvested and then frozen at −20 °C prior to further use.

### 2.2 IgG4 isolation

The CCS containing B72.3 IgG4 was allowed to thaw on ice, centrifuged at 4601*g* for 10 minutes and then passed through a 0.45 μm Supor Acrodisk syringe filter to remove large particulate matter. The filtered media was desalted using a Hiprep 26/10 column (GE Healthcare Life Sciences, Little Chalfont, UK) in equilibration buffer (50 mM phosphate/150 mM NaCl, pH 7.4). The desalted media was loaded onto a Protein A MabSelect SuRe column (Cytiva, UK) equilibrated with equilibration buffer. The column was washed with equilibration buffer and bound material eluted with 0.1 M sodium citrate, pH 3.3. The purified IgG4 was eluted into neutralization buffer (1 M Tris-HCl, pH 9.0) to raise the pH. The fractions containing the mAb were pooled and the purified cB72.3 IgG4 fractions were buffer exchanged into equilibration buffer and concentrated using 100 kDa molecular weight cut off filters (Merck Millipore, Billerica, MA, USA). The protein was concentrated to 10 mg ml^−1^. The amount of cB72.3 IgG4 was quantified by OD_280 nm_ using a Nano drop lite (Thermo, Wilmington, DE, USA) with an *E*^1^% of 13.7. Samples were stored at −80 °C for further use.

### 2.3 Static binding capacity measurements

Resin samples analysed in this study were MabSelect SuRe. Protein A affinity purification resin samples from various positions in an AXIChrom 981 ml column previously used for 25 cycles of purification, were provided by GSK Biopharm Process Research. Samples were extracted from different spatial column locations during column unpacking. Precise distances from the column inlet are provided in ESI Table 1.† An aliquot of 20.8 μl of each resin sample was individually packed, equilibrated and dispensed into a 96 well Supor filter (0.45 μm) plate using a MediaScout® ResiQuot (ATOLL, Weingarten, Germany) provided by GSK. Purified B72.3 IgG4 was thawed and diluted to give a range of concentrations (1, 2, 3, 4, 5, 6 and 7 mg ml^−1^) in equilibration buffer. Diluted IgG4 (200 μl) was individually added to the resin samples, and the resin + protein samples mixed (1000 rpm) for 45 minutes at room temperature. The concentration of unbound IgG4 that flowed through the packed resin was analysed at OD_280 nm_ on a Nano drop lite with an *E*^1%^ = 13.7. Resin samples were stored at 4 °C. Unused MabSelect SuRe was used as a control.

The Langmuir adsorption isotherm was used to determine the mAb binding capacity of the resin samples from different locations within the pilot scale column. The measured amount of protein in the flow-through (*C*_eq_) after loading samples of various concentrations of IgG4 (*C*_0_) was used to calculate the binding capacity (*Q*) of different resin samples using eqn (1):^30^1
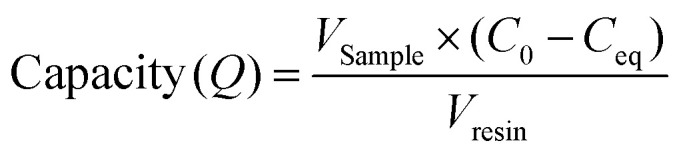


Once the binding capacities of individual resin samples were calculated, the data was fitted to a Langmuir isotherm (2). This allows for prediction of maximum binding capacity (*Q*_max_) and dissociation constant (*K*_d_) for each resin sample.2
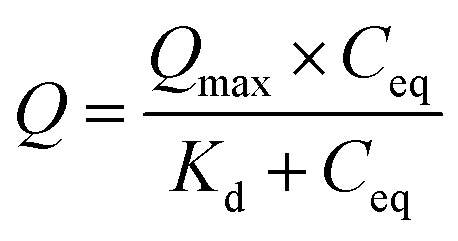


### 2.4 ATR-FTIR spectroscopic measurements of spent Protein A resin samples after SBC experiments

**Fig. 1:**
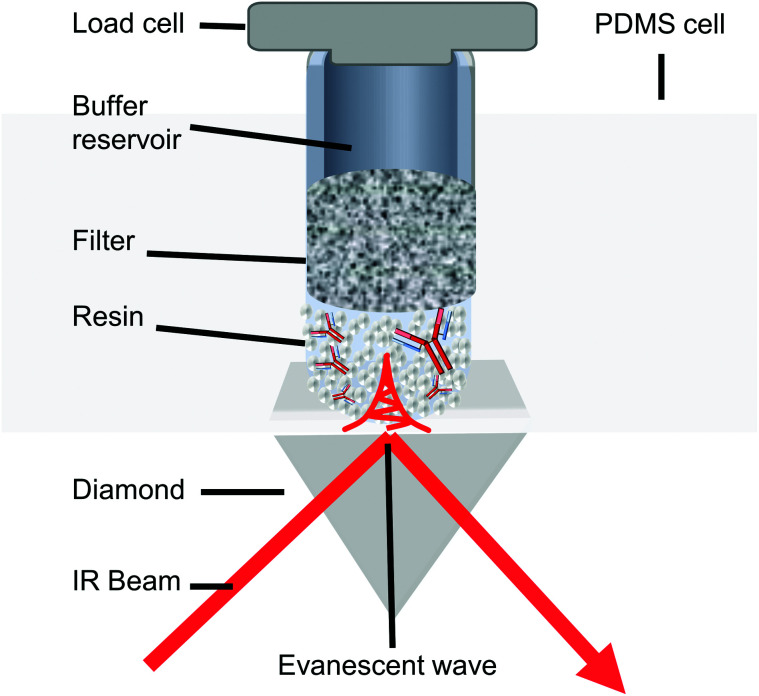
Schematic of the *in situ* ATR-FTIR spectroscopic setup used for analysis of the Protein A resin samples. The PDMS microwell device was affixed to the ATR accessory by an acrylic top plate. The resin was pressed against the diamond IRE with a controlled load monitored by the load cell. The plunger contained a porous filter and buffer reservoir to ensure small sample volumes did not dry out during measurements. Note that this schematic is not to scale. The evanescent wave is much smaller than the resin in reality.

A 1.3 mm custom PDMS microwell device was attached to a custom-cut PMMA housing and affixed to a Diamond Golden Gate™ ATR accessory (Specac, Orpington, UK). The accessory was used with an Equinox 55 FTIR spectrometer (Bruker, Germany) equipped with an MCT detector. Static binding capacity resin samples (≈10.4 μl in 10 μl of 50 mM PBS, 150 mM NaCl, pH 7.4) were loaded into the microwell. The microwell contents were subjected to a 200 g load through a polyethene filter tip, and custom cut 19-gauge plunger (Fig. 1). Samples were then measured by co-adding 64 scans between 3800 cm^−1^–800 cm^−1^ at a spectral resolution of 1 cm^−1^.

ATR-FTIR spectra were collected using OPUS 5.5 (Bruker, Germany). Single channel spectra were ratioed using PBS buffer background spectra and a built-in atmospheric compensation algorithm (utilized simulated vapour spectra). This ensured all background buffer liquid and vapour was removed from the spectra. The removal of spectral bands of water was confirmed by the absence of the libration + OH bending mode.^31^ The generated absorption spectra were then imported to Orange^32^ with Quasar addon.^33^ A rubber band baseline correction was applied to the ATR-FTIR spectra in the range 1800.0 cm^−1^–853.6 cm^−1^ using Orange. For PLS quantification, ATR-FTIR spectra were normalized at the glycosidic bending mode of the agarose base matrix at 1067 cm^−1^. All subsequent data analysis was performed in MATLAB (MathWorks, Natick, USA).

### 2.5 PLS quantification of mAbs bound to Protein A resin

SBC analysis was used to measure the binding capacity of unused MabSelect SuRe generating a total of 23 data sets. PLS regression (PLSR) of the ATR-FTIR spectral data sets obtained for these unused MabSelect SuRe samples, using Q as the regression target, were utilised to generate a PLS predictive method. The number of PLS components was chosen based on the lowest root-mean-squared error after Leave One Out Cross Validation (LOOCV) (ESI Fig. 1†). Three PLS components were chosen. The model was then applied to the ATR-FTIR spectral data sets of the spent resin (a total of 6 spectra for each resin sample) that had been saturated with IgG4 (loaded with 5, 6 and 7 mg ml^−1^).

### 2.6 Local Protein A quantification

A Protein A standard curve (ESI Fig. 2†) was generated using recombinant Protein A (Merck, Gillingham, UK) plotting the OD_280 nm_ readings obtained on a Nano drop Lite with *E*^1%^ = 1.65 ref. (34) against ATR-FTIR integrated absorbance of the amide II band (1482–1590 cm^−1^). A range of Protein A concentrations between 0 and 52.95 mg ml^−1^ were used. This standard curve was used to quantify the local Protein A concentration of the GSK spent resin samples with no mAbs bound based on the absorbance of the amide II band obtained in each case.

## 3.Results and discussion

### 3.1 Used MabSelect SuRe performance at different spatial locations

**Fig. 2:**
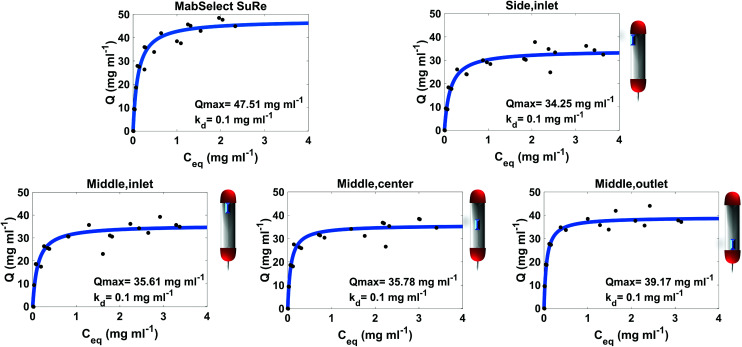
Static binding capacity of unused MabSelect SuRe and resin samples from different defined locations within a used MabSelect SuRe column. Three spent samples were assessed from the middle (inlet, center and outlet) and one spent sample from the Side (inlet) of the column. The approximate locations of the resin samples in the original column are indicated by the schematics next to the individual plots. Spent resin has undergone 25 purification cycles. Each OD_280_ nm measurement was carried out in duplicate.

The adsorption isotherm plots generated provide the *Q*_max_ and *K*_d_ of each resin sample from the column (Fig. 2). As expected, the unused MabSelect SuRe exhibited the highest *Q*_max_ value at 47.51 mg ml^−1^. All the used resin samples exhibited a decrease in *Q*_max_ compared to the unused resin. The greatest loss in static binding capacity compared to the unused resin is seen in the resin samples at the inlet of the column, where the CCS would be loaded for purification. Resin harvested from the middle outlet of the column retained a higher *Q*_max_ of 39.17 mg ml^−1^. The sample from the center of the column had a slightly higher but overall very similar *Q*_max_ to the inlet samples. On average, the used column exhibited a *Q*_max_ decrease to 36.20 mg ml^−1^ compared to 47.51 mg ml^−1^ for unused MabSelect SuRe (Table 1).

**Table 1:** Quantified adsorbed mAb and local Protein A ligand concentrations on PrAc resin

Sample	*Q*_max_ a (mg ml^−1^)	Local Protein A concentration^b^ (mg ml^−1^)	Average binding capacity^c^ (mg ml^−1^)	Average binding capacity prediction^d^ (mg ml^−1^)
MabSelect SuRe	47.51 ± 2.07	31.30 ± 9.9	43.38	N/A
Middle, inlet	35.61 ± 4.47	32.73 ± 12.1	34.41	25.80
Middle, center	35.78 ± 1.48	27.63 ± 0.6	33.78	36.10
Middle outlet	39.17 ± 2.6	20.37 ± 7.4	38.01	32.72
Side, inlet	34.25 ± 2.37	29.04 ± 1.7	32.71	28.28
Overall column	36.20 ± 2.73	29.77 ± 5.45	34.63	30.08

a *Q*_max_ values obtained from static capacity binding measurements.

b Local protein A concentration values obtained from ATR-FTIR spectroscopy measurements of resin samples. Amide II band was integrated.

c Average binding capacity is the average binding capacity (Q) of resin when saturated with antibodies by loading with C0 of 5, 6 and 7 mg ml^−1^ IgG4.

d Average binding capacity prediction is the predicted binding capacity of saturated samples obtained from PLSR analysis.

**Fig. 3:**
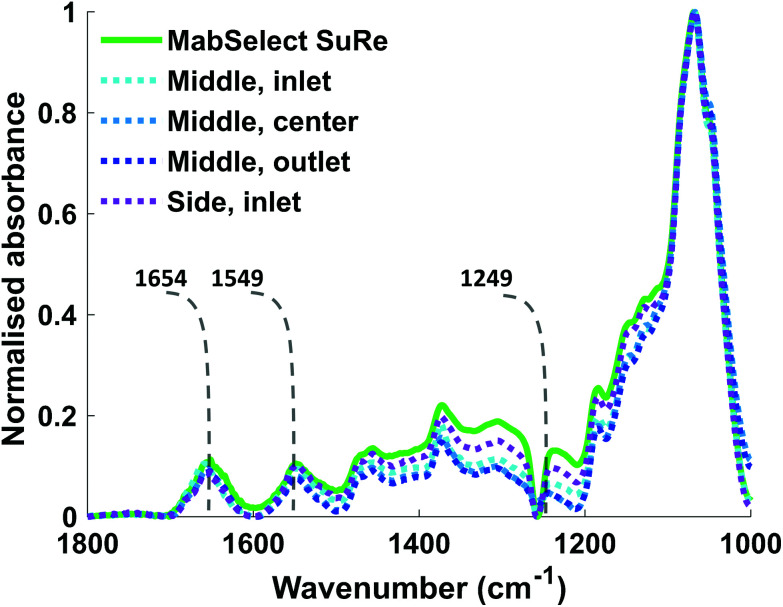
ATR-FTIR spectra of unused MabSelect SuRe and used MabSelect SuRe from different column locations. Spectra in the range between 1800 cm^−1^–1000 cm^−1^ of different resin samples with labelled amide I, II & III bands at 1654 cm^−1^, 1549 cm^−1^ and 1249 cm^−1^ respectively. There are no significant changes in the amide I and II bands of the resin samples measured.

The *K*_d_ of mAb binding to the Protein A ligand was the same (0.1 mg ml^−1^) for each of the test samples as well as the MabSelect SuRe control, indicating that resin use and resin location within the column do not affect binding affinity. This lack of change in binding affinity indicates that despite 25 purification cycles of use the Protein A is not structurally altered. This finding is supported by ATR-FTIR spectroscopy, as the Protein A ligand spectral bands appear at the same wavenumber in both the unused MabSelect control and the used resin samples (Fig. 3). ATR-FTIR spectroscopy of all resin samples show the amide I band at 1654 cm^−1^, indicative of a primarily alpha helical protein,^27^ in agreement with the known crystal structure of Protein A.^35^

Our SBC data clearly show that the effects of repeated use on resin differs according to the location of the resin within the column, with resin at the column inlet exhibiting lower binding capacity than that at the outlet. These findings are in agreement with another study on ion exchange columns.^36^ Whilst SBC analysis reports on the reduction in the ability of the column to bind antibody it doesn't provide information on the changes within the column that account for this reduction.

### 3.2 PLS analysis of ATR-FTIR spectroscopic measurements of used Protein A resin to predict spatial location performance

**Fig. 4:**
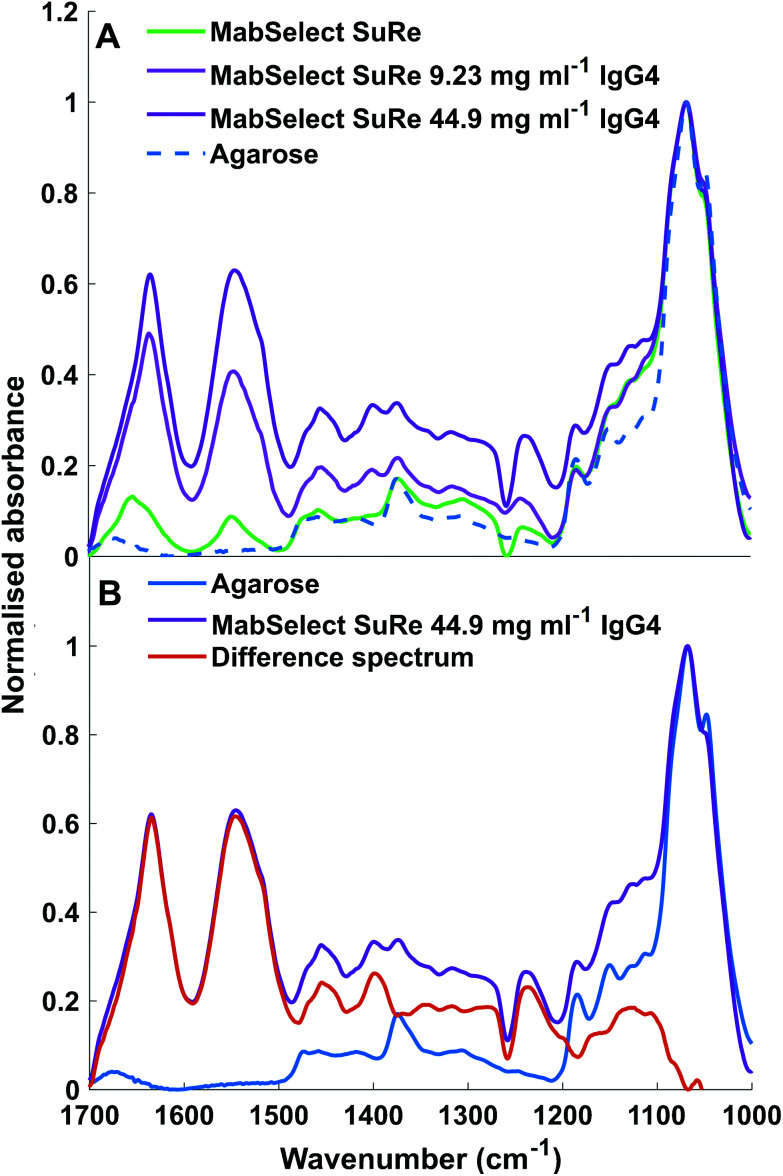
(A) ATR-FTIR spectra of unused MabSelect SuRe in the absence of bound mAbs (green) and mAbs bound at 9.23 mg ml^−1^ (light purple) and 44.90 mg ml^−1^ (dark purple) as well as non-functionalised agarose (dotted blue). (B) ATR-FTIR spectra of Agarose and MabSelect Sure with difference spectrum. MabSelect SuRe with 44.90 mg ml^−1^ IgG4 adsorbed (dark purple), non-functionalised agarose (blue), difference spectrum of MabSelect SuRe with 44.9 mg ml^−1^ IgG4 and agarose (orange). Spectral bands between 1200–1500 cm^−1^ can are clearly revealed as specific to the mAb by the difference spectrum.

ATR-FTIR spectroscopy is sensitive to both chemical functional groups and protein secondary structure of measured samples. This sensitivity can be observed when comparing the spectra obtained for unused MabSelect SuRe control resin with MabSelect SuRe resin bound to different concentrations of mAb (9.23 mg ml^−1^ and 44.90 mg ml^−1^). Peaks between 1200 cm^−1^ and 1500 cm^−1^ are masked by peaks representative of the base agarose matrix (Fig. 4). We revealed these bands by subtraction of the agarose base matrix spectrum allowing for band assignment in this region. As shown in Table 2 and Fig. 4, a wide range of bands present in the measured spectra are representative of the bound mAb and thus can be used for quantification.

**Table 2:** Band assignment of MabSelect SuRe resin with adsorbed mAbs in the spectral region 1700 cm^−1^–1000 cm^−1^

Wavenumber (cm^−1^)	Band assignment
1688 (shoulder)	Amide I β sheets – protein A
1654 (shoulder)	Amide I α helix – protein A^27^
1634 (main peak)	Amide I β sheets – IgG4^25,37^
1545 (main peak)	Amide II unordered – IgG4^27^
1540 (shoulder)	Amide II unordered – IgG4^27^
1519 (shoulder)	Amide II – IgG4^37^
1455	CH_3_*δ*_as_ – IgG4^25,37^
1398	CH_3_*δ*_s_ – Valine and Alanine – IgG4^28^
1375	Glycosidic linkage – polysaccharide^38^
1238	Amide III β sheets-IgG4^25,37^
1185	Glycosidic linkage – polysaccharide^38^
1150	Glycosidic linkage – polysaccharide^38^
1067	Glycosidic linkage *ν* – polysaccharide^38^

Partial least squared (PLS) analysis allows utilization of all bands the algorithm detects as being related to the *Y* value, in this case the capacity, *Q* (eqn (2)) of mAbs bound to the resin sample. This data contains arrays of thousands of observations representing the absorbance at different wavenumber (1800.0 cm^−1^–853.6 cm^−1^) for each resin sample with known concentrations of mAb bound. The data is broken down into components with each component representing a % of variance in the *y* value. The training data set utilized here was the *in situ* ATR-FTIR spectra of unused MabSelect SuRe Resin samples obtained from the OD_280 nm_ static binding capacity experiments. In total there were 24 spectra used, with 1 spectrum excluded due to poor IRE contact (ESI Fig. 3†). The training data set utilized 3 components which explained 84.09% of *Y* variance in the training data. The test data consisted of spectra obtained from ATR-FTIR spectroscopic measurements of 36 spent resin samples saturated with mAbs, with 5 spectra excluded due to poor IRE contact (ESI Fig. 3†). The optimum number of PLS components used was chosen based on the lowest root mean squared error (RMSE) of the cross-validation set. LOOCV was used to test the model.

**Fig. 5:**
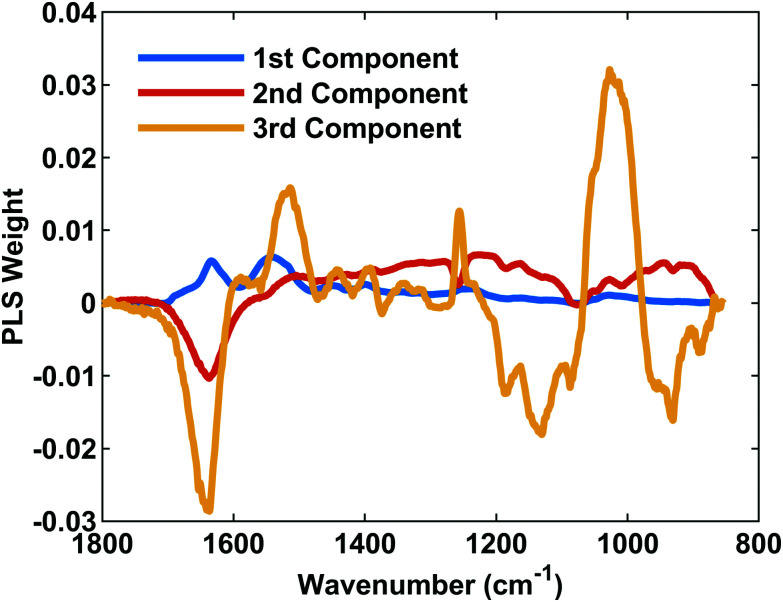
PLSR loading plot. The loading plot indicates the spectral bands representative of each component used with the 1^st^, 2^nd^ and 3^rd^ components shown in blue, red and yellow respectively. The 1^st^ component is representative of most variance in the training spectra, followed by the 2^nd^ and 3^rd^ respectively. A strong PLS weight means a band represents more of the PLS component. Amide I and II bands vary most with varying mAb concentration.

The loading plot spectra generated by the training data set of unused MabSelect SuRe (Fig. 5) shows the weighting of spectral peaks for each component used to quantify the amount of absorbed mAb. We found that component 1 was predominantly made up of the amide I and II bands at 1634 cm^−1^ and 1540 cm^−1^ respectively, with these peaks accounting for 72.77% variance in mAb concentration. Adding a 2^nd^ component predominantly made up of the amide II band shoulder at 1519 cm^−1^ and amide III band at 1234 cm^−1^ accounted for a total of 80.87% of the variance in mAb concentration. The third component, representative of the amide II band as well as β sheet CH_3_ bending and alanine/Valine bending at 1452 cm^−1^ and 1400 cm^−1^ respectively, contributed just an extra 3.22% of variance. This component also selected two peaks represented by non mAb components; PDMS Si–CH_3_ at 1256 cm^−1^ and the C–OH Agarose peak at 1008 cm^−1^. The total variance provided by all three components corresponds to 84.09%.

The average binding capacity of saturated spent PrAc resins obtained from the PLS analysis shows the same trend as *Q*_max_ values with the resin obtained from the inlet of the used column having the lowest capacity and the resin obtained from the outlet retaining the highest capacity. We found that the RMSE when applying our model to the prediction of used resin was 6.14 mg ml^−1^ (Table 3). To compare against the traditional approach for measuring SBC, the coefficient of variation (%CV) of the RMSE of our ATR-FTIR PLS model was calculated as 18%:3
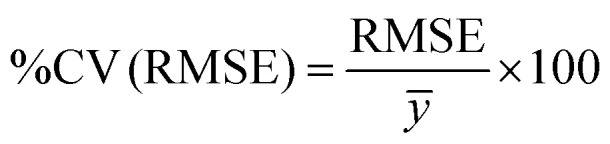
*ȳ* is the average actual measurement.

**Table 3:** Statistical analysis of the PLS model with 3 components. Observed vs fitted concentrations of mAbs bound to PrAc resin samples (ESI Fig. 4†) were used to assess the PLS model. Observed data is the Q value calculated from SBC assays and fitted is the prediction from the PLS model

Data set	Statistic	Value
Training	*R* ^2^	0.84
	RMSE	6.08 mg ml^−1^
LOOCV	*Q* ^2^	0.75
	RMSE	7.84 mg ml^−1^
Test	*Q* ^2^	0.12
	RMSE	6.14 mg ml^−1^

This was then compared to the %CV calculated for the SBC assay for the overall column average binding capacity; 11%.4
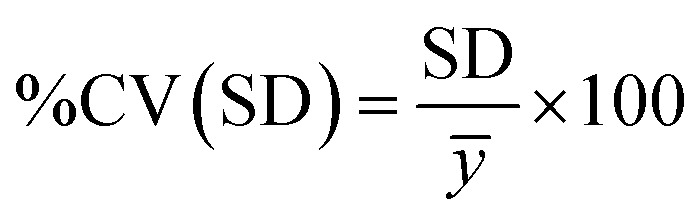


Our model produces a good predication of the binding capacity of Protein A resin, meeting industry standards (<20%) and providing additional molecular information when compared to the SBC assay.

PLSR analysis of ATR-FTIR spectroscopic data allowed for the use of multiple spectral bands representing mAbs bound to used PrAc resins, ensuring an accurate prediction of the binding capacity. As expected, the PLS component explaining the most variance in mAb concentration was made up of the Amide I and Amide II bands, as these are the most informative bands for determining secondary structure from the absorption spectra of proteins.

Our data clearly demonstrate that *in situ* ATR-FTIR spectroscopy can be used to accurately assess the overall performance of a used column. With PLS regression analysis we predicted the overall binding capacity of the used column, when saturated with mAbs, to be 30.08 mg ml^−1^. This prediction is just 4.55 mg ml^−1^ below the SBC calculated binding capacity of 34.63 mg ml^−1^. Intriguingly, the side inlet exhibited the greatest reduction in binding capacity both from the SBC measurements and the ATR-FTIR spectroscopic analysis, with a slightly lower *Q*_max_ than the middle inlet. The resin at the side of the column is subjected to a phenomenon known as the wall effect, which results in resin being less packed at this location, forming a preferential route of flow in the column. A potential consequence of this is that resin at the side of the column is exposed to more contaminants/larger build-up of irreversibly bound mAb than in the centre of the column.^39^

### 3.3 Quantification of Protein A in the spent resins

Previous research from our group^20^ and Pathak *et al.*^40,41^ has indicated loss of Protein A ligand as a reason for reduction in SBC. Thus, using *in situ* ATR-FTIR spectroscopy we quantified the local Protein A concentration on each resin sample. This is possible because the absorbance of the spectral bands of proteins in measured ATR-FTIR spectra is proportional to protein concentration. The amide II band was chosen for quantification as it does not overlap with the water bending mode band at ≈1640 cm^−1^, unlike the amide I band at ≈1654 cm^−1^. The local Protein A concentration we obtained for MabSelect SuRe of 31.30 mg ml^−1^ is higher than the resin average of 5.6 mg ml^−1^ stated by manufacturers.^42^ This high local concentration compared to the resin average is likely caused by the shallow probing depth^43,44^ of the evanescent wave (effective thickness, <4 μm at 1600 cm^−1^) utilized in ATR-FTIR spectroscopy.^20^ In our case, the local Protein A concentration is calculated from ATR-FTIR spectra representative of the layer of Protein A molecules adjacent to the IRE, and thus the local concentration is not the total amount of Protein A present in the sample, as previously described.^20^ However, it is in this work that used resins from the different regions of a pilot-scale industrial affinity column were studied.

The local Protein A concentration of the used resin samples did not significantly vary from each other or the unused MabSelect SuRe (Table 1), indicating that the loss in binding capacity is not due to Protein A leaching. There was no correlation between local Protein A concentration and static binding capacity (*Q*_max_) measurements of the different resin samples. This finding, together with the spectroscopic analysis revealing no detectable changes in secondary structure of the Protein A ligand (no shift or alteration in shape of the Amide I, II and III bands, Fig. 3) in the used resin samples compared to control, indicated that Protein A ligand loss and denaturation are not causes of the reduction in binding capacity observed for the used resin samples. It is possible that the reduction in SBC is caused by fouling of the resin by either host cell proteins/DNA or the build-up of irreversibly bound mAb.^18^ This is supported by another study which indicated that lower binding capacity at the inlet of an ion exchange column, as seen here for a Protein A column, was the result of greater fouling due to the load material contact time being the highest here.^36^

## 4. Conclusions

In summary, using a combination of *in situ* ATR-FTIR spectroscopy and SBC assays we show that the reduction in PrAc static binding capacity is heterogeneous throughout a used pilot scale chromatography column, with the greatest loss of binding capacity occurring at the column inlet. This could allow for further use of the PrAc resin at a column's outlet helping to reduce the cost of mAb purification. Currently, column performance is assessed by overall column binding capacity and not by a detailed analysis of different regions of a column.

Importantly we reveal that this loss of SBC is not due to Protein A ligand leaching or denaturation. Our data rather suggest that the reduction in binding capacity is due to irreversible fouling. The chemical nature of these contaminants remains to be revealed. The contaminants are not directly observable in this study, likely due in part, to these being under the limit of detection after just 25 cycles of purification. Lintern *et al.* reported significant contaminant build up, as detected by MS/MS, after 80 cycles of purification.^18^ In addition, another study reported that fouling tends to occur in the centre of PrA resin beads.^36^ The penetration depth of the evanescent wave used for ATR-FTIR only probes ∼5 μm of the beads, which can be up to 120 μm in diameter indicating that this technique is unlikely to detect contaminants bound to these resin samples. Since ATR-FTIR spectroscopy is more sensitive to surface layer proteins and less able to probe the interior of the MabSelect SuRe beads, further analysis using confocal Raman microscopy, which can probe further into the beads, might provide additional insights into the causes of binding capacity loss.

This study demonstrates the power inherent in *in situ* ATR-FTIR spectroscopy, as the molecular information gained from this approach allows quantification of mAb binding, assessment of Protein A ligand concentration and Protein A ligand conformation. Thus the use of *in situ* ATR-FTIR spectroscopy in this research represents a substantial advance over SBC analysis alone, providing an in-depth assessment of why resin samples exhibit reduced binding capacity. Therefore, this approach may have a significant potential in industrial mAb processing settings.

## Author contributions

Conceptualization BB and SGK; Methodology JB; Original Draft JB; Writing-Review and Editing BB, SGK, JB, RRJ and MF; Funding Acquisition BB, SGK, RRJ; Resources RRJ, MF and RT; Supervision BB and SGK.

## Conflicts of interest

There are no conflicts to declare.

## Supplementary Material

AN-146-D1AN00985K-s001
