# Supplementary material for: Insight into purification of monoclonal antibodies in industrial columns via studies of Protein A binding capacity by in situ ATR-FTIR spectroscopy
Source: Analyst. 2021 Jul 16;146(16):5177–85. doi: 10.1039/d1an00985k (PMC8351638; doi:10.1039/d1an00985k)
Supplement: AN-146-D1AN00985K-s001 [file AN-146-D1AN00985K-s001.pdf]

Electronic Supplementary information (ESI)

Insight into purification of monoclonal antibodies in industrial columns via studies of Protein A binding capacity by *in situ* ATR-FTIR spectroscopy

James W. Beattie[a,b] Ruth C. Rowland-Jones[c], Monika Farys[c], Richard Tran[c], Sergei G. Kazarian[b*], Bernadette Byrne[a*]

a. Department of Life Sciences, Imperial College London, South Kensington Campus, SW7 2AZ, London, UK.

b. Department of Chemical Engineering, Imperial College London, South Kensington Campus, SW7 2AZ, London, UK.

c. Biopharm Process Development, GlaxoSmithKline, Gunnels Wood Road, Stevenage, Hertfordshire SG1 2NY, UK.

To whom correspondence should be addressed: s.kazarian@imperial.ac.uk and : b.byrne@imperial.ac.uk

Please do not adjust margins

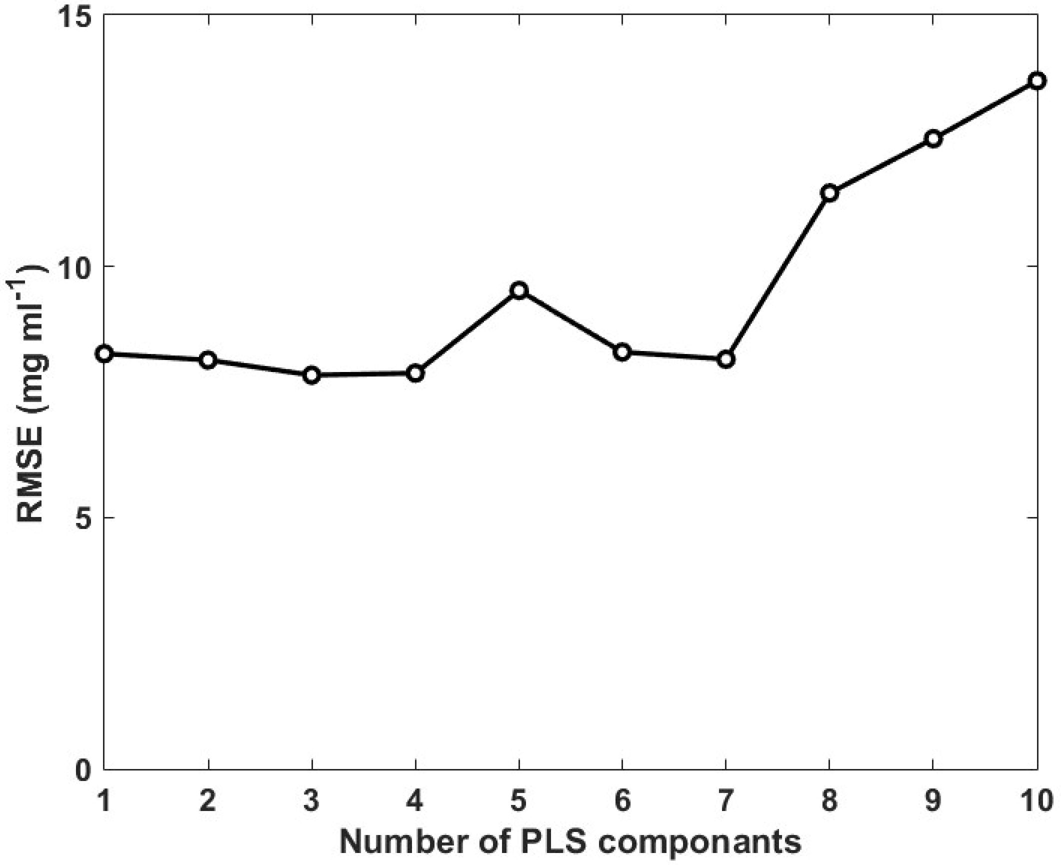

Figure 1 Root mean squared error of PLS model with increasing number of PLS components. RMSE was calculated by plotting fitted vs observed model for each component using LOOCV (Leave one out cross validation). A total of three PLS components was shown to be optimal for this model.

This journal is © The Royal Society of Chemistry 20xx

Please do not adjust margins

Please do not adjust margins

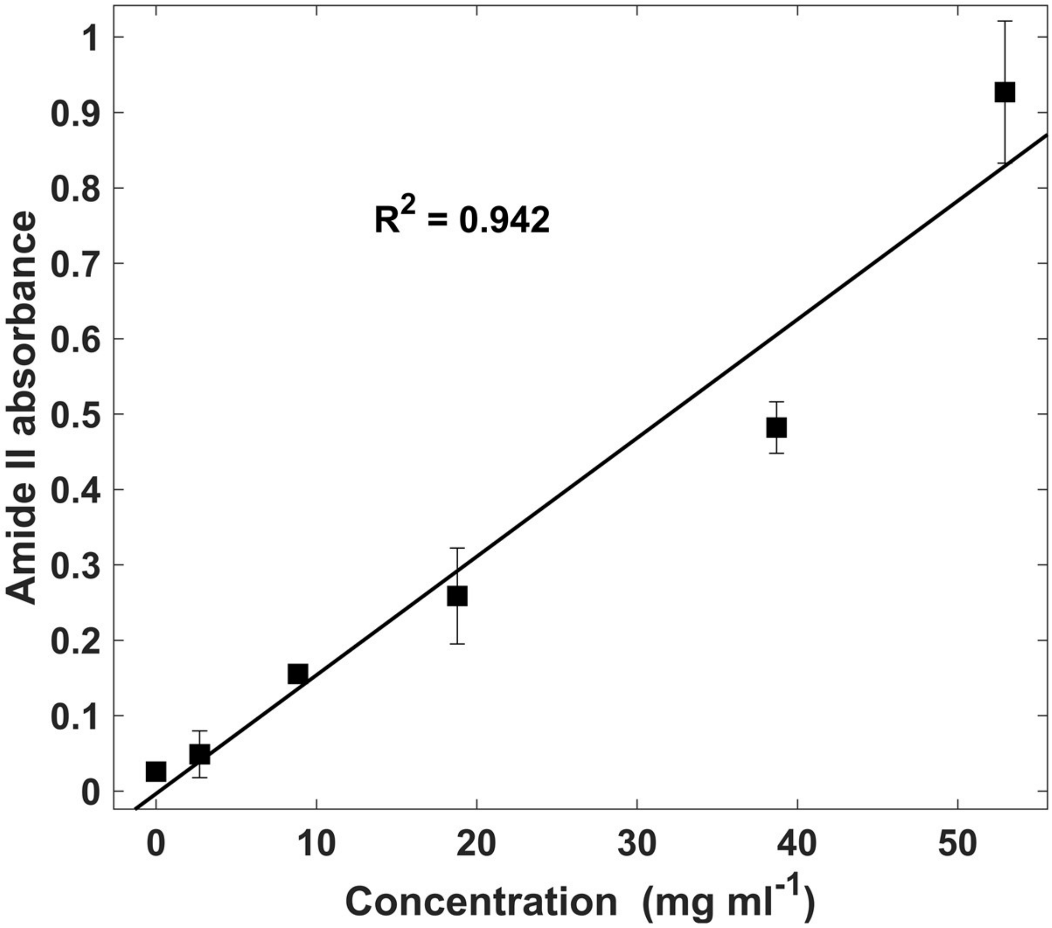

Figure 2 Protein A calibration curve. Amide II absorbance integrated between 1590^{-1} and 1482 cm^{1} was plotted against known protein A concentrations calculated by using Beer-lambert law (OD= 280 nm, $E^{1\%}$=1.65). The data shown is an average of n=2 independent ATR-FTIR spectroscopy experiments.

This journal is © The Royal Society of Chemistry 20xx

Please do not adjust margins

Please do not adjust margins

Table 1 Location of resin samples used in study and their respective k_d and Qmax values. The column positions are measured in distance vertically from the column inlet. K_d and Q_{max} are calculated from the SBC assay.

Sample	Distance from column inlet (cm)	K_d (mg ml^{-1})	Q_{max} (mg ml^{-1})
MabSelect SuRe	N/A	0.12	47.51
Middle, inlet	0 - 5	0.1	35.61
Middle, centre	12-17	0.1	35.78
Middle, outlet	21 - 26	0.1	39.17
Side, inlet	0 - 2	0.1	34.25

This journal is © The Royal Society of Chemistry 20xx

Please do not adjust margins

Please do not adjust margins

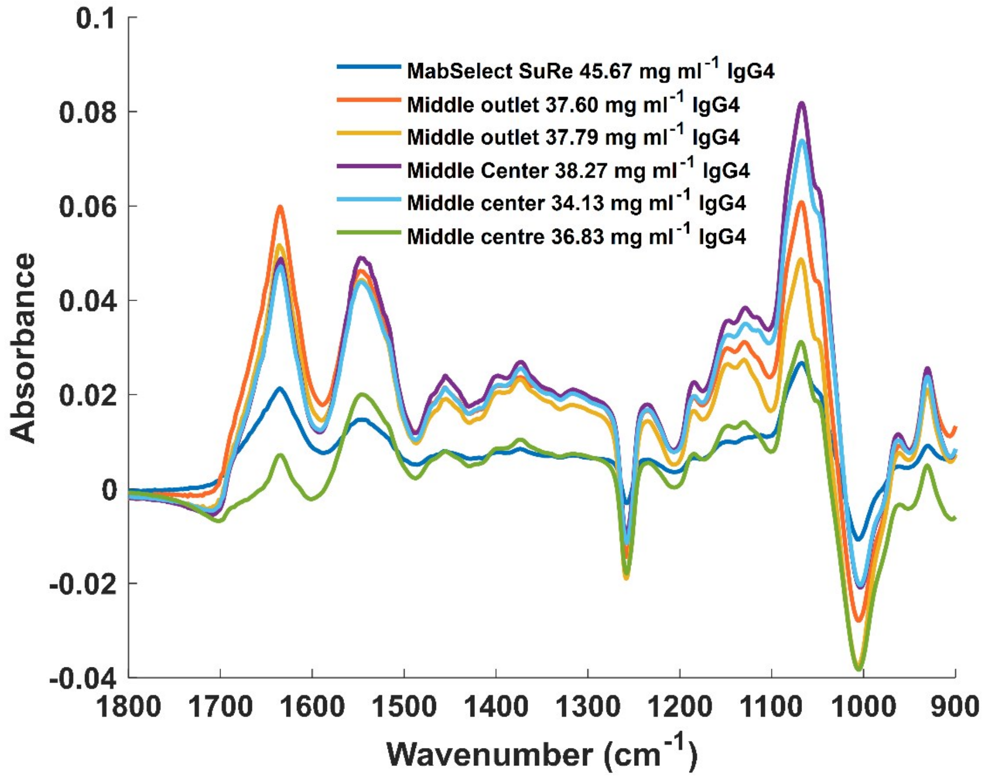

Figure 3 ATR-FTIR spectra excluded from the PLS training and test data. Test data was evaluated by comparing prediction to SBC observed binding capacities, if values were over 10 mg ml^{-1} different then raw spectra was assessed. MabSelect SuRe 45.67 mg ml^{-1} IgG4 (dark blue) was removed from the training data due to poor contact as shown by extremely weak glycosidic bending agarose band at 1067 cm^{-1}, upon normalisation this would cause Amide bands to be overrepresented. The middle center 36.83 mg ml^{-1} IgG4 (green) also exhibited weak agarose band. Middle Centre 36.83 mg ml $^{-1}$ (green) exhibited overly weak Amide I band. The Amide I should be the same as or higher than the Amide II band due to extra contributions from the C=O stretch. This was due to over subtraction of Water bending mode which results in under predictions of resins samples. Middle outlet 37.60 mg ml^{-1} (orange) IgG4 exhibited strong subtraction of PDMS bands at 1256 cm^{-1}, this is due to PDMS from the microwell device being replaced by the resin beads as more PDMS is present in background spectrum compared to single channel. Middle outlet 37.79 mg ml^{-1} (yellow), Middle center 38.27 mg ml^{-1} (purple) IgG4 and middle center 36.83 mg ml $^{-1}$ (green) IgG4 also exhibited PDMS being displaced by resin. This results in underrepresented bands after normalisation and weakened bands in the 1000-1500 cm^{-1} range.

Please do not adjust margins

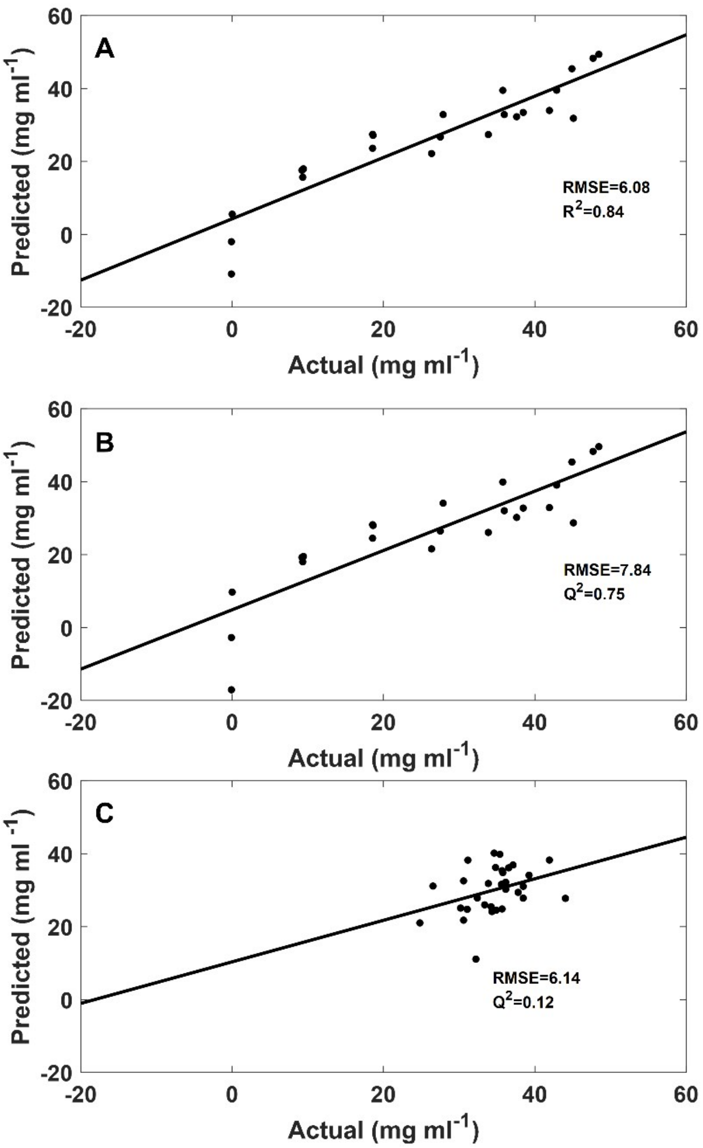

Figure 4 Actual vs predicted plot concentrations of mAbs bound to the PrAc resin samples. A) Training data, B) leave one out cross validation and C) test data. Actual data is the Q value calculated from the SBC assays and predicted data was obtained from the PLS model.

 This journal is © The Royal Society of Chemistry 20xx
